# Aflatoxin B_1_ invokes apoptosis via death receptor pathway in hepatocytes

**DOI:** 10.18632/oncotarget.14158

**Published:** 2016-12-24

**Authors:** Muhammad Jameel Mughal, Xi Peng, Yi Zhou, Jing Fang

**Affiliations:** ^1^ Key Laboratory of Animal Diseases and Environmental Hazards of Sichuan Province, College of Veterinary Medicine, Sichuan Agricultural University, Chengdu, Sichuan, PR China; ^2^ Key Laboratory of Southwest China Wildlife Resources Conservation (Ministry of Education), College of Life Sciences, China West Normal University, Nanchong, Sichuan, China; ^3^ Life Science Department, Sichuan Agricultural University, Yaan, Sichuan, PR China

**Keywords:** aflatoxin B1, apoptosis, carcinogenicity, oxidative stress, hepatotoxicity

## Abstract

The fungal metabolites produced by *Aspergillus flavus* and *Aspergillus parasiticus* cause detrimental health effects on humans and animals. Particularly aflatoxin B_1_ (AFB_1_) is the most studied and a well-known global carcinogen, producing hepatotoxic, genotoxic and immunotoxic effects in multiple species. AFB_1_ is shown to provoke liver dysfunctioning by causing hepatocytes apoptosis and disturbing cellular enzymatic activities. In liver, AFB_1_ causes apoptosis via extrinsic mechanism because of high expression of death receptor pathway. The detailed mechanism of AFB_1_ induced hepatocytes apoptosis, via death receptor pathway still remains elusive. So the present study was conducted to explore apoptotic mechanism initiated by death receptors and associated genes in aflatoxin B1 induced liver apoptosis in chickens fed with AFB_1_ for 3 weeks. Results from the present study displayed histopathological and ultrastructural changes in liver such as hydropic degeneration, fatty vacuolar degeneration and proliferation of bile duct in hepatocytes in AFB_1_ group, along with imbalance between reactive oxygen species (ROS) and antioxidant defense system upon AFB_1_ ingestion. Moreover, AFB_1_ intoxicated chickens showed upregulation of death receptors FAS, TNFR1 and associated genes and downregulation of inhibitory apoptotic proteins XIAP and BCL-2. The results obtained from this novel and comprehensive study including histopathological, ultrastructural, flow cytometrical and death receptor pathway gene expression profiles, will facilitate better understanding of mechanisms and involvement of death receptor pathway in hepatocytes apoptosis induced by AFB_1_ and ultimately may be helpful in bringing down the toxigenic potential of AFB_1_.

## INTRODUCTION

Aflatoxins are probably the most intensively researched mycotoxins in the world as 4.5 billion people worldwide suffer from intemperate exposure to aflatoxins which causes 4.6–28.2% of all global hepatocellular carcinoma cases [1, 2]. These fungal metabolites are mainly produced by *Aspergillus flavus* and *Aspergillus parasiticus*. The most common aflatoxins are B_1_, B_2_, G_1_ and G_2_ which are naturally present in many food products and affect more than one organ system simultaneously, therefore producing cascade of responses in the affected organism [3, 4]. Among the major aflatoxins, aflatoxin B1 (AFB_1_) is the most widely known carcinogen [5] having highly hepatotoxic, genotoxic, immunotoxic, and other adverse health effects on humans and several other animal species [6]. International Agency for Research on Cancer (IARC) have produced sufficient evidences of carcinogenicity of AFB_1_ and classified it as a Group 1 human carcinogen and intended no safe dose [7].

As the major drug metabolizing and detoxifying organ in the body, liver is primarily affected followed by ingestion of the aflatoxins [8, 9]. AFB_1_ is potent hepatocarcinogen in humans and exposure to AFB_1_ is known to cause both chronic and acute hepatocellular injury. AFB_1_ action mechanism involves DNA adducts formation and mutation at codon 249 of the p53 tumor suppressor gene leading to carcinogenicity in humans [10–12]. Although AFB_1_ are hepatotoxic, causing pallor discoloration, enlargement, congestion and necrosis of liver along with triggering proliferation of bile duct and infiltration of mononuclear and heterophilic cells in many livestock and laboratory animals, yet susceptibility varies with breed, species, age, dose, length of exposure and nutritional status [13–15].

Apoptosis is a programmed form of cell death [16, 17] and many studies have demonstrated its role in implicating pathogenesis of multiple diseases in humans and animals [18–20]. It is also well documented in different model systems that aflatoxins react antagonistically with different cell proteins, leading to inhibition of carbohydrate and lipid metabolism and protein synthesis, which can induce apoptosis [21]. Liao *et al*., 2014, investigated that AFB_1_ can provoke liver dysfunctioning by promoting hepatocytes apoptosis and disturbing cellular enzymatic activities. AFB_1_ induced apoptosis can be initiated by two main apoptotic pathways: the extrinsic and the intrinsic. Although intrinsic pathway is also involved in different tissue apoptosis [22, 23], yet in liver cells due to high level of death receptor expression, apoptosis predominantly occurs via the extrinsic pathway [24] and contributes to the development of a number of liver diseases including liver cirrhosis, cholestasis, viral hepatitis and hepatocellular carcinoma [25, 26]. Our previous studies have revealed that AFB_1_ negatively affect the spleen, thymus, jejunum and ilium and elucidated the mechanisms by which AFB_1_ induces apoptosis in these organs and tissues in chicken [27–31]. Looking at the earlier reports in different organ systems, it would be enthralling to find out how liver is affected at cellular and molecular level, by AFB_1_. In order to understand this, the present study is designed to explore the apoptotic mechanisms primed by death receptor molecules in AFB_1_ induced liver apoptosis by analyzing histological, ultra structural, biochemical, flow cytometrical and relative expression changes in apoptosis-regulating genes in hepatocytes using chicken as a research model, considering the fact that chicks are susceptible to AFB_1_ exposure [32]. As cell proliferation and apoptosis are directly related to carcinogenicity [33], the outcomes from present study could facilitate the understanding of AFB_1_ induced carcinogenicity and may be helpful in bringing down the toxigenic potential of AFB_1_.

## RESULTS

### Histological and ultrastructural analysis

**Figure 1:**
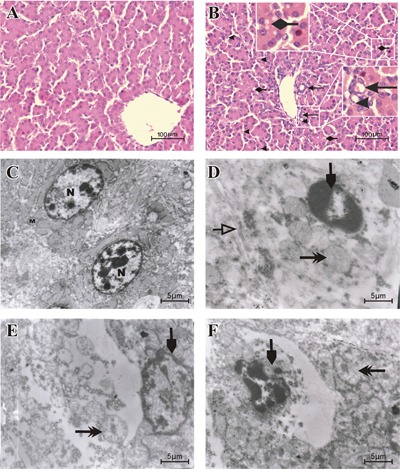
Histopathological and Ultrastructural changes displayed by liver cells when exposed to AFB1 Histological examination of H & E stained liver tissues from 21 days old chickens fed on control diet **A**. and AFB_1_ **B**. (Bar = 100 μm). Proliferation of bile duct←. Fragmented nuclei◂. Hydropic and vacuolar fatty degeneration←. Ultrastructural examination of uranyl acetate and lead citrate stained liver tissue from the control group C. with normal nucleus (N), normal mitochondria (M) and other normal organelles and the AFB1 group D, E and F (Bar = 5 μm). Condensed, irregular or fragmented nucleus (↓, D, E and F), swollen mitochondria (↠, D, E and F) with decreased cristae (➩, E) and swollen endoplasmic reticulum (➩, D).

Microscopically, degenerative reversible lesions were seen, from mildest to severest degree with various distributions in AFB_1_ group. Compared to control group (Figure 1A), slight to moderate hydropic degeneration, fatty vacuolar degeneration and proliferation of bile duct in hepatocytes were seen in AFB_1_ group. Moreover pyknotic and fragmented nuclei were also visible in AFB_1_ group (Figure 1B). Results of ultrastructural observation revealed irregular, fragmented and condensed nucleus, swollen mitochondria with reduced number of cristae and swollen endoplasm reticulum in AFB_1_ group ( Figure 1D-1F) when compared to control (Figure 1C). AFB_1_ induced histopathological lesions were observed in chicken liver. The incidence of liver congestion, vacuolar or fatty degeneration, proliferation of bile duct and nuclear fragmentation were seen going up from day 7 to day 21 in AFB_1_ intoxicated chickens whereas no lesions were observed in the control group (Table 1).

**Table 1:** Incidence of major histopathological lesions of chicken liver

Pathological lesions	Time	Control group	AFB_1_ group
	7 days	0/6	1/6
**Liver congestion**	14 days	0/6	2/6
	21 days	0/6	4/6
	7 days	0/6	1/6
**Vacuolar or fatty degeneration**	14 days	0/6	3/6
	21 days	0/6	5/6
	7 days	0/6	2/6
**Proliferation of bile duct**	14 days	0/6	4/6
	21 days	0/6	6/6
	7 days	0/6	1/6
**Increased nuclear fragmentation**	14 days	0/6	2/6
	21 days	0/6	5/6

Incidence of histopathological lesions in the liver among chickens from different experimental groups (n=6)

### Apoptotic percentage by flow cytometer

**Figure 2:**
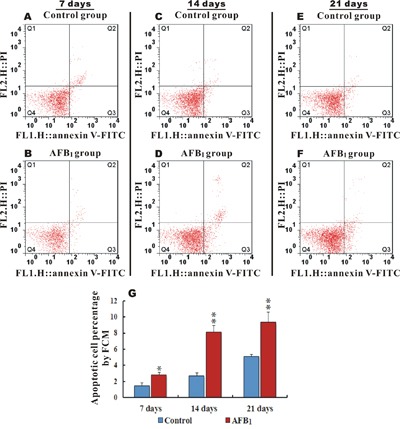
Apoptotic hepatocytes percentage and scattered analysis of early and late AFB1 induced apoptosis in chicken hepatocytes Figure **A,C,E,** and **B, D, F.** represents scattered analysis of early (Annexin-V positive and PI negative) and late (Annexin-V and PI positive) hepatocytes apoptosis in the control and AFB_1_ groups at 7, 14 and 21 days respectively, while **G.** is a bar graph showing apoptotic percentage rate in the control and AFB_1_ group at day 7, 14 and 21 by flow cytometer (number of chickens, n=6). *p<0.05, or **p<0.01, when compared to the control.

FITC Annexin V was used to quantitatively determine the percentage of cells within groups that were actively undergoing apoptosis. Apoptotic cell counts were determined by examining the total percentage of early (Annexin-V positive and PI negative) and late (both Annexin-V and PI positive) apoptotic cells. Figure 2A and 2B, 2D, 2F shows scattered analysis of early and late hepatocytes apoptosis at 7, 14 and 21 days in the control and AFB_1_ groups respectively. When compared to the control, the AFB_1_ group at 7, 14 and 21 days displayed a significantly increased percentage of apoptotic cells (*p*<0.05 or *p*<0.01) (Figure 2G).

### Biochemical analysis

**Figure 3:**
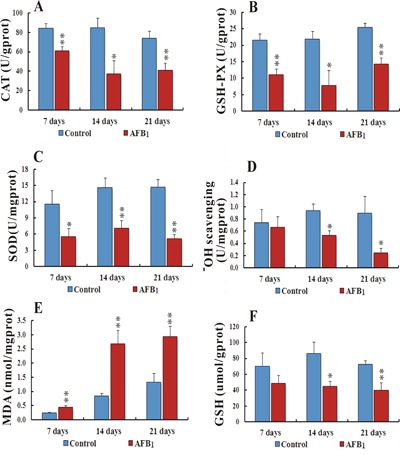
Biochemical analyses of activities of CAT, GSH-Px, SOD, Hydroxyl ion scavenging and contents of MDA and GSH in liver cells intoxicated with AFB1 Figure **3A-3C,** represents activities of CAT, GSH-Px and SOD respectively. Bar graph D. shows Hydroxyl free radical scavenging while **E** and **F**. bar graphs display contents of MDA and GSH in chicken hepatocytes (number of chickens, n = 6). *p<0.05, or **p<0.01, when compared to the control.

Compared to the control group, the activities of CAT (Catalase) and GSH-Px (Glutathione Peroxidase) in the AFB_1_ group were observed significantly downregulated (*p*<0.05 or *p*<0.01) throughout the experiment at different time points. Activity of SOD (Superoxide Dismutase) and Hydroxyl free radical scavenging were also seen remarkably down (*p*<0.05 or *p*<0.01) in the AFB_1_ group compared to the control at 14 and 21 days. The contents of MDA (Malondialdehyde) were observed significantly upregulated at 7, 14 and 21 days (*p*<0.01), while GSH (glutathione) contents were seen reduced (*p*<0.05 or *p*<0.01) at 14 and 21 days in AFB_1_ group when compared to the control group (Figure 3).

### qRT-PCR analysis of relative expression of genes involved in death receptors induced apoptosis

**Figure 4:**
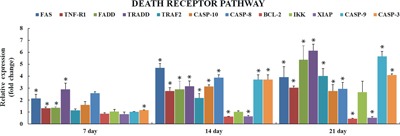
Relative expression of genes involved in death receptor induced apoptosis in liver of chickens exposed to AFB1 Figure 4 represents the mRNA levels of FAS, TNF-α, FADD, TRADD, TRAF2, CASPASE 10, CASPASE 8, BCL-2, IKK, XIAP, CASPASE 9 and CASPASE 3 in liver of the AFB_1_-fed chickens and expressed as fold change relative to the control group (number of chickens, n = 6). *p<0.05, or **p<0.01, when compared to the control.

The death receptor genes FAS (Fatty acid synthetase receptor) and TNF R1 (Tumor necrosis factor receptor 1), and the genes involved in DISC (death-inducing signaling complex) formation i-e FADD (FAS-associated death domain), TRADD (TNF receptor-associated death domain), and TRAF2 (TNF receptor-associated factor 2) were seen significantly upregulated (*p*<0.05 or *p*<0.01) at 7, 14, & 21 days with an exception of TRAF2 which was not significantly upregulated at day 7 in AFB_1_ group when compared with the control group. The mRNA levels of CASPASE (cysteine-aspartic protease) family genes, i-e. CASPASE 3, CASPASE 8, CASPASE 9, and CASPASE 10 were seen significantly raised at 14 and 21 days (*p*<0.05 or *p*<0.01), also CASPASE 3 was seen significantly upregulated at day 7 in AFB_1_ group compared to the control. BCL-2 (B-cell lymphoma 2) and XIAP (X-linked inhibitor of apoptosis protein) genes displayed notable downregulation (*p*<0.05 or *p*<0.01) at day 14 and 21, while mRNA levels of IKK (IκB kinase) did not change throughout 7, 14 and 21 days, although it was seen increasing at day 21 but not significant enough (*p*>0.05) (Figure 4).

## DISCUSSION

In the current study we scrutinized the mechanisms of AFB_1_ induced hepatocyte apoptosis by judging the involvement of apoptosis- associated genes and oxidant situation. The histopathological changes in AFB_1_ intoxicated livers were in general agreement with previous reports [34, 35]. Slight to moderate hydropic degeneration, fatty vacuolar degeneration, proliferation of bile duct, massive congestion in midzonal areas, pyknotic and fragmented nuclei within hepatocytes were seen in AFB_1_ intoxicated chickens. Furthermore various electron microscopically detectable changes characterized apoptosis, such as condensed and hyperchromatic chromatin, fragmented nuclei, swollen mitochondria and swollen endoplasmic reticulum, which were in concordance with previously published work by several research groups [16, 19, 36]. The flow cytometric analysis revealed increased hepatocyte apoptotic percentage in AFB_1_ group, which was similar to our previous findings in thymus, spleen, intestine and bursa of fabricius [23, 30, 31, 37] and this increased hepatocyte apoptosis can possibly be associated with AFB_1_ induced carcinogenicity since the rates of cell proliferation and cell death are directly related to cancer [32]. To understand the involvement of apoptosis associated genes in liver cells, we further explored the expression of death receptor signaling pathway involved in hepatocyte apoptosis.

Induction of apoptosis can be triggered by the activation of death receptors including FAS, TNF-α, followed by DISC (death-inducing signaling complex) formation, which consists of CASPASE 8, and associated proteins such as FADD, TRADD and TRAF2 [17]. The present study revealed over expression of FAS and TNF-R1 receptors along with their associated genes i-e CASPASE 8, CASPASE 10, FADD, TRADD and TRAF2, which was consistent with earlier reports in human hepatocytes [38, 39] and with our recent study on thymocytes apoptosis [23]. IKK is crucial in activation of NF-kB based intracellular survival signaling and can activate the expression of several anti-apoptotic proteins [40, 41]. XIAP is apoptotic inhibitor that can plug up the process of cell death induced by any foreign stimuli [42]. In our present study mRNA levels of IKK were not seen significantly increased throughout the experiment at different time points and XIAP expression was significantly decreased at 14 and 21 days and the expression of FADD was much higher than TRAF2. Taken all together present study insinuated that TNF1-TRADD mediated signaling is originating from TRADD, supported by ability of FADD to activate apoptosis rather than activating NF-kB cell survival signaling.

**Figure 5:**
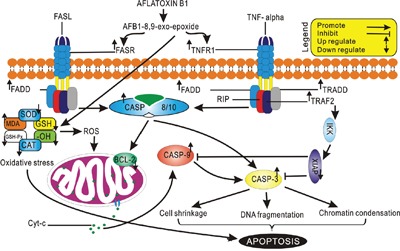
Schematic representation of Aflatoxin B1 induced apoptosis through Death Receptor Signaling

The CASPASE-cascade system plays vital roles in the induction, transduction and amplification of intracellular apoptotic signals [43] and overexpression of CASPASE family genes can force cells to undergo apoptosis. In present study, AFB_1_ administration led to overexpression of CASPASE family genes (CASPASE 10, 8, and 3), suggesting that apoptosis induced by FAS and TNFR1 activates CASPASE 8, which contains an N-terminus with FADD, so providing a direct link between cell death receptors and the Caspases. In addition overexpression of CASPASE 9 and down-regulation of BCL-2 genes indicated that mitochondrial pathway was also involved in hepatocyte apoptosis induced by AFB_1_ (Figure 5). These results were in agreement with previous studies in human and rat hepatocytes [44–47] and our recent report in thymus apoptosis induced by AFB_1_ [23].

Recently many studies have focused on revealing the pivotal role of ROS as well as resulting oxidative stress in inducing apoptosis [48, 49]. It's also previously been demonstrated that disequilibrium between ROS formation and antioxidant defense system may cause DNA damage and mitochondrial disintegration by producing oxidative stress which could lead to apoptosis [50–52]. Oxidative damage is relieved and free radicals are removed from the system by crucial catalyst, such as SOD, CAT and GSH-Px. O2 is converted in to H2O2 by SOD, which is further converted in to H2O by GSH-Px [53]. MDA is an important index of antioxidant ability, since it is the main product of lipid peroxidation [54]. In present study, activities of CAT, GSH-Px and SOD were observed significantly decreased in AFB_1_ treated group along with hydroxyl ion free radical scavenging. A significantly increased contents of MDA and reduced contents of GSH were seen in AFB_1_ treated group. These results were constant with previous studies in multiple organs in different species [23, 30, 50, 55–57]. These results clearly demonstrated that AFB_1_ could cause imbalance between ROS formation and antioxidant defense by accumulating ROS and producing oxidative stress that could play an important role in mediating hepatocytes apoptosis.

## MATERIALS AND METHODS

All the chickens and AFB_1_ experiments have been conducted in accordance with the ethical standards, declaration of Helsinki and national and international guidelines have been approved by Sichuan Agricultural University Animal Care and Use Committee (Approval No: 2012-024).

### Chickens and diets

One hundred and fifty-six one-day-old healthy Cobb-500 broilers were purchased from Chia Tai Group (Wenjiang, Sichuan, China), and were randomly divided into two groups which are the control group (0 mgAFB_1_/kg of basal diet) and AFB_1_ group (0.6 mg AFB_1_/kg of basal diet) with 26 chickens in each group and each group in triplicates. The basal diet, which was control diet, was formulated according to National Research Council (NRC, 1994) [58] and Chinese Feeding Standard of Chicken (NY/T33-2004) recommendations. The AFB_1_-contaminated diet was prepared, using the method described by Kaoud [59]. Briefly, 27 mg AFB_1_ (A6636, Sigma-Aldrich, USA) was dissolved into 30 ml methanol, and the solution was mixed with 45 kg corn-soybean basal diet to formulate the AFB_1_-contaminated diet. The control diet was prepared using the same constituents except AFB_1_. Then the methanol of diets was evaporated at 98 °F (37 °C). The AFB_1_ concentrations were analyzed by HPLC (Waters, Milford, MA, USA) with fluorescence detection (Waters, Model 2475, Milford, MA, USA), and the AFB_1_ concentration was determined as <0.001 mg/kg and 0.601 mg/kg respectively in the control diet and AFB_1_ diet. chickens were housed in cages with electrically heated units and provided with water as well as aforementioned diet *ad libitum* for 21 days.

### Histopathological and ultrastructural examination

At the age of 7, 14 and 21 days, six chickens in each group were euthanized and the livers were fixed in 4% paraformaldehyde (PFA) and routinely processed in paraffin. Thin sections (5 μm) of tissue were sliced, mounted on glass slides and stained with hematoxylin and eosin Y. The histological organization of the tissues were contemplated and snapped with a digital camera (Nikon, eclipse 50i, Japan).

In each group one chick per replicate, was euthanized and then immediately necropsied at the end of the trial. Small pieces of liver tissues were immediately fixed with 2.5% glutaraldehyde and post-fixed in 2% Veronal acetate-buffered OsO_4_. The tissues were embedded in Araldite after dehydrating in alcohol gradient. The blocks were sectioned in 65-75 nm thick sections in a microtome with a glass knife and placed in uncoated copper grids. The sections were stained with uranyl acetate, and post-stained with 0.2% lead citrate. The subcellular architecture of liver was examined with a Hitachi H-600 transmission electron microscope (Japan).

### Annexin v apoptosis detection by flow cytometry

At 7, 14, and 21 days of the experiment, six chickens in each group were euthanized, and livers were sampled from each chick to determine the percentage of apoptotic cells by flow cytometer, using the method by Chen et al. 2011 [60]. Briefly, the dissected livers were thereupon homogenized to form a cell suspension and filtered, and then the cells were washed and resuspended in phosphate buffer at a concentration of 1x 10^6^ cells/mL. 5 μL Annexin V-Fluorescein isothiocyanate (V-FITC) and 5 μL propidium iodide (PI) were added into 100 μL cell suspension, and incubated at 25 °C for 15 min in the dark. 400 μL 1 x Annexin binding buffer was added to the mixture, and then the apoptotic cells were assayed by flow cytometer (BD FACSCalibur) within 1 h. The annexin V-FITC Kit was obtained from BD Pharmingen (USA, 556547).

### Quantitative real-time PCR (qRT-PCR)

Livers from six chickens in each group were removed at 7, 14, and 21 days of age, and instantly stored in liquid nitrogen. The livers samples were homogenized in liquid nitrogen, by crushing with a mortar and pestle and the powdered tissues were collected into eppendorf tubes and stored at -80°C. Total RNA was extracted using TriPure Isolation Reagent kit (Cat No. 11667165001, Roche Applied Science, Germany) following manufacturer's protocol. The quality and quantity of total RNA was measured spectrophotometrically. Extracted RNA was forthwith reverse-transcribed into cDNA using Transcriptor First Strand cDNA Synthesis Kit (Cat No: 04897030001, Roche Applied Science, Germany). qRT-PCR reactions were performed in a total volume of 20 μL using FAStStart Universal SYBR Green Master mix (Cat No: 04913914001, Roche Applied Science, Germany), at the following thermocycler program; Initial denaturation at 95 °C for 10 min, followed by 44 cycles of “10 s at 95 °C and 30 s at melting temperature (T_m_) of a specific primer pair”, and melt curve analysis by 10 s at 95 °C, and 72 °C for 10 s, using Thermal Cycler (Step One Plus, Applied BioSystems, USA). β-actin was used as an internal control [61, 62]. Primers information is provided in Table 2. The qRT-PCR data were analyzed and fold change in expressions were calculated using 2^-ΔΔCt^ calculation method described by [63].

**Table 2:** List of oligonucleotides used as primers in qRT-PCR analysis

Gene symbol	RefSeq mRNA number	Forward primer	Reverse primer	Amplicon length (bp)
**FAS**	NM_001199487	TCCACCTGCTCCTCGTCATT	GTGCAGTGTGTGTGGGAACT	78
**TNF-R1**	NM_001030779	CCTGTCTGTCTTCCCTGTCC	GGTGCATGGGGTCTTTTCTA	120
**TRADD**	XM_414067	CTAGAGCCCAAAGGAAGTCGAT	TGGCTGCTTCTCTGTGACAT	100
**FADD**	XM_421073	GGGGTAAAGAGGCTGAACTCTTA	TGAGTCCTATTGCACTGCTGTC	163
**TRAF2**	XM_015279623	CGTGGTGATGAAAGGACCCA	AATGATGTGCTCCCGGTTGT	100
**Casp-10**	XM_421936	CTGGGGGCTCCAAAAGTCC	AAAGGGGGACAAAGCCAACA	204
**Casp-9**	AY057940	CCAACCTGAGAGTGAGCGATT	GTACACCAGTCTGTGGGTCGG	87
**Casp-8**	NM_204592	GTCTCCGTTCAGGTATCTGCT	TCTCAATGAAAACGTCCGGC	143
**Casp-3**	NM_204725	TGGCCCTCTTGAACTGAAAG	TCCACTGTCTGCTTCAATACC	139
**IKK (IKBIP)**	XM_001232182	GGCTTGGTTTTGGCAGTGAG	CGGCTTTGACGTTTGCTGAA	144
**XIAP**	NM_204588	GCAGAATATGAGAGGCGGATAC	TCCTTCCACTCTTGCAATCC	149
**BCL-2**	NM_205339	TGTTTCTCAAACCAGACACCAA	CAGTAGGCACCTGTGAGATCG	205
**β-actin**	L08165	TGCTGTGTTCCCATCTATCG	TTGGTGACAATACCGTGTTCA	178

### Biochemical analysis

Six chickens in each group were euthanized and immediately necropsied at 7, 14 and 21 days of age. Then livers were immediately taken out and chilled to 0°C in 0.85% NaCl, and then dried, weighed and homogenized in 9 vol of icecold 0.85% NaCl in a chilled homogenizer and centrifuged at 3500 × g at 4°C for 10 min. Total protein was measured by the method of Bradford [33] The commercial kits were purchased from Nanjing Jiancheng Bioengineering Institute (Nanjing, China) and used to detect total protein (Total protein quantification kit No.A045), activities of SOD (Superoxide dismutase detection kit No.A001-1), CAT (Catalase detection kit No.A007), GSH-Px (Glutathione peroxidase detection kit No.A005) and contents of GSH (Glutathione detection kit No.A006) and MDA (Malonaldehyde detection kit No.A003-1), and ^−^OH radical (Hydroxyl free radical detection kit No.A018) in the supernatant, according to the manufacturer's instructions.

### Statistical analysis

The significance of difference between two groups was analyzed by variance analysis, and results are expressed as the mean value with deviation. The analysis was performed using the independent sample *t* test of SPSS software for Mac v.20.0 (IBM Corp, Armonk, NY, USA) and a value of *p*<0.05 was considered significant, while *p*<0.01 was considered markedly significant.

## CONCLUSION

Tissue specific apoptosis caused by AFB_1_ induced toxicity has been studied in much detail, yet the mechanisms of death receptor induced apoptosis in hepatocytes remain least explored. In present study, we have delineated the mechanisms involved in death receptor provoked apoptosis using chicken liver as a model system. The results obtained in this study could be further used to understand involvement of death receptors in apoptosis and could help in amending the toxic effect provoked by AFB_1_.

## Abbreviations

AFB_1_: aflatoxin B1
CAT: Catalase
GSH-Px: Glutathione Peroxidase
SOD: Superoxide Dismutase
MDA: Malondialdehyde
GSH: glutathione
FAS: Fatty acid synthetase receptor
TNF R1: Tumor necrosis factor receptor 1
DISC: death-inducing signaling complex
FADD: FAS-associated death domain
TRAF2: TNF receptor-associated factor 2
CASPASE: cysteine-aspartic protease
BCL-2: B-cell lymphoma 2
XIAP: X-linked inhibitor of apoptosis protein)
IKK: IκB kinase
